# C1-C2 Transarticular Anterior Fixation for the Treatment of Atlantoaxial Traumatic Dislocation: A Clinical Case

**DOI:** 10.1055/s-0042-1748812

**Published:** 2022-06-20

**Authors:** Paulo Gil Ribeiro, Francisco Fernandes, Paulo Costa, Ana Catarina Quintas, Paulo Lourenço, Diogo Lino Moura

**Affiliations:** 1 Setor de Coluna Vertebral, Serviço de Ortopedia, Centro Hospitalar e Universitário de Coimbra EPE, Coimbra, Portugal; 2 Faculdade de Medicina, Universidade de Coimbra, Coimbra, Portugal

**Keywords:** axis cervical vertebra, geriatrics, odontoid apophysis, osteosynthesis, spinal fractures

## Abstract

Fractures of the odontoid apophysis are one of the most frequent lesions in the elderly population, and an increasingly preponderant problem with the progressive aging of the world population.

In the present work, we report a clinical case of an 88-year-old male patient who suffered a fall resulting in a type-II fracture of the odontoid apophysis on the Anderson-D'Alonzo classification. Given the age and comorbidities of the patient, we decided to perform osteosynthesis of the fracture through anterior fixation with a transarticular screw in combination with fixation with an odontoid screw.

This technique enables the necessary stability for the consolidation of Anderson-D'Alonzo's type II odontoid apophysis fracture, with the advantage of the lower levels of dissection of the cervical extensor musculature and hemorrhage resulting from this aggression when compared with the posterior approach; moreover, it is a readily-available technique that yields clear benefits in the treatment of this pathology in the geriatric population.

## Introduction

Fractures of the odontoid apophysis contribute 9% to 15% of all cervical spine lesions in adults, and they are the most prevalent cervical lesion among the elderly. 1 With the aging of the population, the absolute number of fractures of the cervical spine has increased, especially C2 fractures. 2

This pathology is usually treated by posterior fixation of C1-C2 through the techniques developed by Magerl and Seeman 3 or Harms and Melcher. 4 Among the main disadvantages of these techniques are the aggressive dissection of the cervical extensor muscles and the abundant hemorrhage that resulting from their use.

Another option to provide C1-C2 stabilization option is the insertion of transarticular screws by the anterior cervical route. 1 5 The concept consists of bilateral transarticular fixation with screws to the lateral masses of C1 and C2, similar to the technique described by Magerl and Seeman; 3 however, in this case, fixation is performed by the anterior route, avoiding the invasiveness of the posterior pathway, potentially accelerating recovery and favoring early mobilization, which is paramount in the geriatric population in order to minimize complications.

Anterior fixation with a C1-C2 transarticular screw was first described by Barbour 6 in 1971 for patients with atlantoaxial instability, and, in 1982, Böhler 7 described the use of anterior screw fixation to the odontoid in the treatment of odontoid fracture.

In the present work, we report a clinical case and the surgical technique used complemented with step-by-step images showing the benefits of anterior fixation with a transarticular screw in combination with odontoid screw fixation for Anderson-D'Alonzo 8 type-II odontoid fractures in the elderly population. 5 9

## Clinical Case

The work was carried out according to the Helsinki Declaration of the World Medical Association on ethical principles for medical research involving human beings, and the patient agreed and signed the free and informed consent form.

**Fig. 1:**
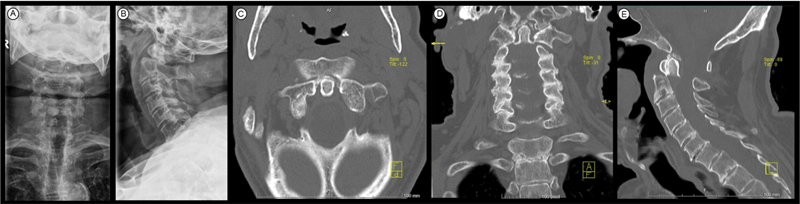
Radiograph and CT scan of the cervical spine - fracture-dislocation of the odontoid apophysis – AP ( **A** ) and lateral incidences ( **B** ), on the axial ( **C** ), coronal ( **D** ), and sagittal planes ( **E** ).

An 88-year-old male patient who had suffered a fall with cranioencephalic trauma was admitted to our hospital, a level-1 trauma center, after being transferred from another facility. After performing computed tomography (CT), a type-II Anderson-D'Alonzo 8 fracture-dislocation of the odontoid apophysis was diagnosed ( Fig. 1 ). The patient had no neurological deficits and cranial skeletal traction was placed to reduce dislocation and the operative time.

**Fig. 2:**
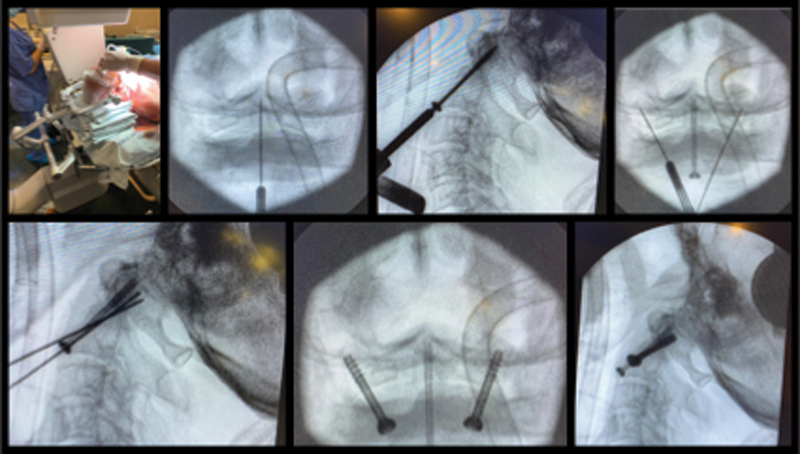
Surgical technique for anterior transarticular C1-C2 fixation in combination with odontoid screw fixation, based on the technique described by Christoph Josten and Ulrich J. Spiegel. 5 9

Considering the patient's age, we decided to perform anterior fixation with a transarticular screw in combination with fixation with an odontoid screw ( Fig. 2 ).

The surgical technique used followed the one described by Christoph Josten and Ulrich J. Spiegel. 5 9 The patient was placed in supine position, and Mayfield cranial traction was applied. Complete fracture reduction was achieved and confirmed by cervical traction fluoroscopy and flexion maneuvers. To maintain the reduction with the necessary cervical flexion, we used towels under the head to support it. We used a roll of surgical sponges to keep the mouth open permanently. Image intensifiers were used, one to obtain an anteroposterior (AP) odontoid view, and the other, for a lateral view of the cervical spine. The level of skin incision is determined with the use of a Kirschner (K) wire, and a cross-sectional incision was made, and the standard approach of the anterior cervical spine was performed. After identifying the correct level, a 2-mm K wire was used and positioned in the center of the odontoid base on AP incidence. In the lateral view, the K wire passes through the C2-C3 intervertebral disc and must be previously positioned on the lower platform of C2. After the correct alignment, we progress until we pierce the cortex at the apex of the tooth. After measurement, a 3.5-mm cannulated screw with short thread and washer was introduced. To achieve bilateral fixation of C1-C2, screws were introduced to the atlantoaxial lateral masses bilaterally. We used 2.0-mm K wires placed about 3 mm to 4 mm laterally to the midline of the lateral odontoid to the tip of the central screw on the AP view and at the same entry point as the screw to the odontoid on the lateral view. The direction is tilted from 20° to 25° laterally in the frontal plane, and from 30° to 40° dorsally in the sagittal plane. The 2.0-mm K wires were inserted into the lateral mass of C1 just below the occipito-cervical joint. Subsequently, two 3.5 mm short-threaded cannulated screws were inserted after measurement.

**Fig. 3:**
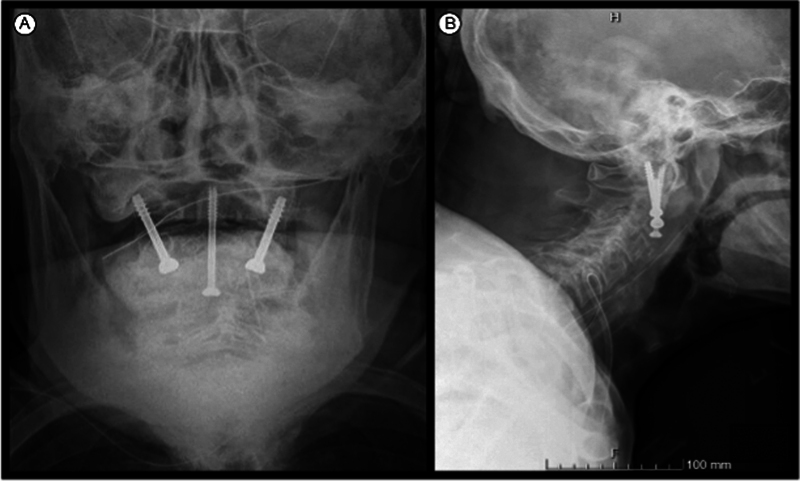
Radiograph of the cervical spine on AP ( **A** ) and lateral ( **B** ) incidences.

**Fig. 4:**
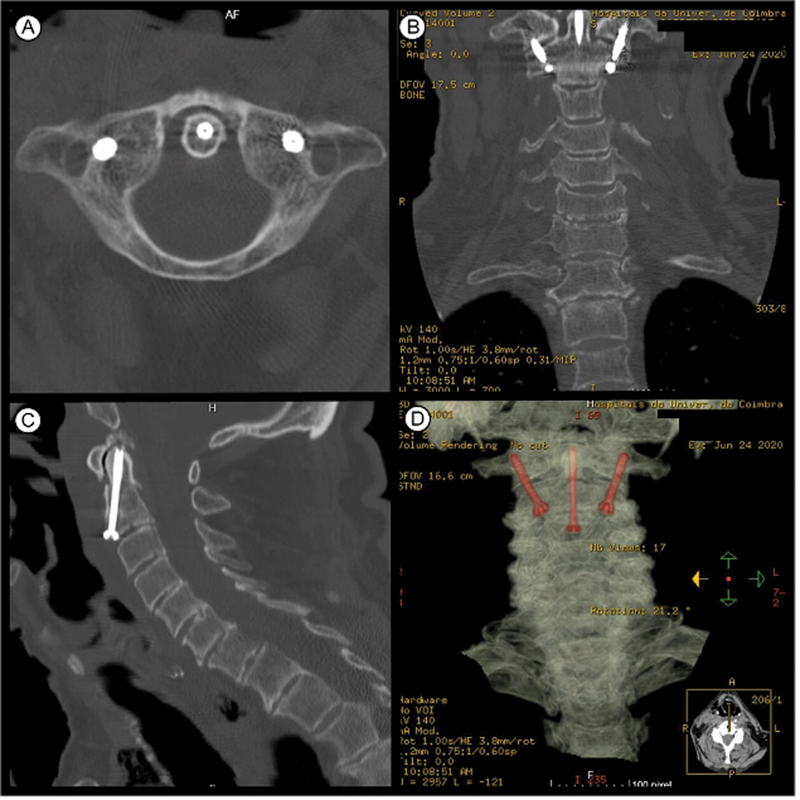
Postoperative CT scan showing correct alignment of the screws on the axial ( **A** ), coronal ( **B** ), and sagittal ( **C** ) planes. Three-dimensional reconstruction ( **D** ).

After surgery, radiographs of the cervical spine and a cervical CT scan were performed to confirm the positioning of the screws ( Figs. 3 and 4 ). A cervical collar was applied for six weeks, followed by two weeks of weening with alternating periods of use.

**Fig. 5:**
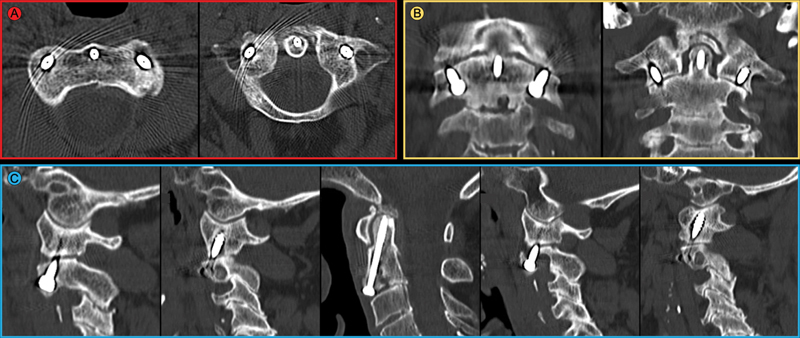
Computed tomography scan at 12 months postoperatively showing fracture consolidation and correct alignment of the screws on the axial ( **A** ), coronal ( **B** ) and sagittal ( **C** ) planes. A slight degree of osteolysis around the transarticular screws can be observed, as expected, because there is no arthrodesis, as mentioned in the discussion.

One year after surgery, the patient is extremely satisfied, without neurological pain or deficits, having returned to the levels of activity prior to the injury. As expected, the patient has had a decrease in cervical rotation, with 35° of rotation to each side. The radiological study at one year shows fracture consolidation ( Fig. 5 ).

## Discussion

Fracture to C2 is the most common cervical lesion in the elderly population, and typé-II fractures on the Anderson-D'Alonzo 8 classification are the most common subtype. In the elderly population, this lesion constitutes 60% to 80% of C2 fractures, which makes this an increasingly prevalent problem due to the progressive aging of the population. 2

In the treatment of Anderson-D'Alonzo 8 type-I and -III fractures, there is a consensus regarding the conservative treatment. However, the treatment of type-II fractures is controversial especially, in the elderly population. 1

For the treatment of this lesion in the elderly population, there is the option of conservative treatment, posterior fusion, or anterior fixation. The conservative treatment is associated with higher rates of pseudoarthrosis and lower quality of life. 5 Posterior atlantoaxial fusion techniques are well established and may yield excellent results, with high fusion rates, but lead to more aggressive surgical dissection and a higher risk of injury to vertebral arteries. The anterior approach benefits from the fact that the patient is in supine position, and the surgical dissection is performed through a virtual space, with lower levels of aggression. 1 5 Anterior fixation is biomechanically comparable to posterior fixation and a valid alternative to this technique. 10

Considering the elderly population, who often has multiple medical commorbidities and high anesthetic risk, lower levels of surgical aggression have clear benefits.

The authors also admit that even if arthrodesis is not accomplished, bilateral transarticular fixation between the C1-C2 lateral masses can guarantee a stable enough fixation, with no reports of failure of these fixations even if arthrodesis is not accomplished. In the geriatric population, we think that this less aggressive fixation technique should be regarded as the first line in cases of traumatic C1-C2 instability. We understand that, in the geriatric population, with fixation with previous transarticular screws, arthrodesis is not necessary, and this stabilization is usually sufficient to ensure good clinical results, with reduced risks of failure. However, precisely due to the absence of arthrodesis, the appearance of some degree of osteolysis is expected, especially around the transarticular screws, as shown in Fig. 5 .

We emphasize that further studies are needed to evaluate the efficacy of this fixation in younger populations, given their higher life expectancy and functional level, and these patients may have a higher risk of failure of these transarticular screws in situations in which there was no evolution to arthrodesis. Thus, we consider this technique ideal for geriatric patients with traumatic C1-C2 instability; however, for this reason, we do not commonly use it in young patients, preferring techniques that include more arthrodesis-favoring gestures, namely posterior fixations associated with cruentation and local application of bone graft. Osteosynthesis of the odontoid apophysis is solely recommended when the fracture line is perpendicular to the osteosynthesis screw, and only in these cases we choose its osteosynthesis. In cases in which the fracture line is not favorable to osteosynthesis, after fracture reduction and C1-C2 dislocation, we opt only for anterior transarticular fixation.
