# Supplementary material for: C1-C2 Transarticular Anterior Fixation for the Treatment of Atlantoaxial Traumatic Dislocation: A Clinical Case
Source: Rev Bras Ortop (Sao Paulo). 2022 Jun 20;59(Suppl 1):e68–72. doi: 10.1055/s-0042-1748812 (PMC11254439; doi:10.1055/s-0042-1748812)
Supplement: Related texts

# Research article: Fixação anterior transarticular C1-C2 para o tratamento de luxação traumática atlantoaxial: Um caso clínico

**Keywords:** apófise odontoide, fraturas da coluna vertebral, geriatria, osteossíntese, vértebra cervical áxis

## Abstract

As fraturas da apófise odontoide são uma das lesões mais frequentes na população idosa, e um problema cada vez mais preponderante com o envelhecimento progressivo da população mundial.

Neste trabalho, apresentamos um caso clínico de um doente do gênero masculino, de 88 anos, que sofreu uma queda da qual decorreu uma fratura da apófise odontoide de tipo II de Anderson-D'Alonzo. Dada a idade e suas comorbilidades, optou-se por realizar a osteossíntese da fratura por meio da fixação anterior com parafuso transarticular em combinação com a fixação com parafuso à odontoide.

Esta técnica que permite a estabilidade necessária para a consolidação da fratura da apófise odontoide de tipo II de Anderson-D'Alonzo, com a vantagem das menores disseção da musculatura extensora cervical e hemorragia decorrente desta agressão quando comparada com a abordagem posterior, sendo uma técnica à disposição e que acarreta benefícios claros no tratamento desta patologia na população geriátrica.

## Introdução

As fraturas da apófise odontoide contribuem com 9% a 15% de todas as lesões da coluna cervical nos adultos, e constituem a lesão cervical mais prevalente na população idosa. 1 Com o envelhecimento da população, o número absoluto de fraturas da coluna cervical tem aumentado, especialmente as de C2. 2

Esta patologia é habitualmente tratada mediante fixação por via posterior de C1-C2 pelas técnicas de Magerl e Seeman 3 ou de Harms e Melcher. 4 Entre as principais desvantagens destas técnicas estão a disseção agressiva da musculatura extensora cervical e a hemorragia abundante delas decorrente.

Outra opção de estabilização de C1-C2 são os parafusos transarticulares, inseridos por via cervical anterior. 1 5 O conceito consiste na fixação transarticular bilateral com parafusos às massas laterais de C1 e C2, à semelhança da técnica descrita por Magerl e Seeman; 3 no entanto neste caso, a fixação é efetuada por via anterior, o que evita a invasividade da via posterior, potencialmente acelera a recuperação, e favorece o levante precoce, algo que é fulcral na população geriátrica de modo a minimizar complicações.

A fixação anterior com parafuso transarticular C1-C2 foi descrita pela primeira vez por Barbour 6 em 1971 para pacientes com instabilidade atlantoaxial, e, em 1982, Böhler 7 descreveu a utilização da fixação anterior do parafuso à odontoide no tratamento da fratura da odontoide.

Neste trabalho, descrevemos um caso clínico e a técnica cirúrgica utilizada complementada com imagens passo a passo que mostram os benefícios da fixação anterior com parafuso transarticular em combinação com a fixação com parafuso à odontoide para as fraturas da odontoide de tipo II de Anderson-D'Alonzo 8 na população idosa. 5 9

## Caso Clínico

O trabalho foi realizado de acordo com a Declaração de Helsinki, da Associação Médica Mundial, sobre princípios éticos para pesquisa médica envolvendo seres humanos, e o paciente concordou e assinou o termo de consentimento livre e esclarecido.

Foi admitido no nosso hospital, um centro de trauma nível 1, transferido de outra instituição hospitalar um doente de 88 anos, do gênero masculino, que tinha sofrido uma queda com traumatismo cranioencefálico. Após realização de tomografia axial computadorizada (TAC), foi diagnosticada uma fratura-luxação da apófise odontoide de tipo II de Anderson-D'Alonzo 8 ( Fig. 1 ). O doente não apresentava défices neurológicos, e foi colocada tração esquelética craniana para reduzir a luxação e temporizar a cirurgia.

**Fig. 1:** Radiografia e TAC da coluna cervical – fratura-luxação da apófise odontoide – nas incidências AP ( **A** ) e lateral ( **B** ), nos planos axial ( **C** ), coronal ( **D** ) e sagital ( **E** ).

Tendo em conta a idade do doente, optou-se por realizar uma fixação anterior com parafuso transarticular em combinação com fixação com parafuso à odontoide ( Fig. 2 ).

**Fig. 2:** Técnica cirúrgica para fixação anterior transarticular C1-C2 em combinação com a fixação com parafuso à odontoide, com base na técnica descrita por Christoph Josten e Ulrich J. Spiegel. 5 9

A técnica cirúrgica utilizada seguiu a técnica descrita por Christoph Josten e Ulrich J. Spiegel. 5 9 O paciente foi colocado em posição supina e foi aplicada a tração craniana de Mayfield. A redução completa da fratura foi obtida e confirmada por fluoroscopia por tração cervical e manobras de flexão. Para manter a redução com a flexão cervical necessária, utilizamos toalhas debaixo da cabeça para a apoiar. Utilizamos um rolo de esponjas cirúrgicas para manter a boca aberta permanentemente. Foram utilizados intensificadores de imagem, um para obter uma vista de odontoide anteroposterior (AP), e o outro, para uma vista lateral da coluna cervical. O nível de incisão da pele é determinado com a utilização de um fio de Kirschner (fio K), e foi feita uma incisão transversal e realizada a abordagem padrão da coluna cervical anterior. Após a identificação do nível correto, foi utilizado um fio K de 2 mm posicionado no centro da base da odontoide na incidência AP. Na vista lateral, o fio K passa pelo disco intervertebral C2-C3 e precisa de ser posicionado anteriormente na plataforma inferior de C2. Após o alinhamento correto, progredimos até perfurarmos o córtex no ápice do dente. Após a medição, foi introduzido um parafuso canulado de 3,5 mm com rosca curta e anilha. Para conseguir a fixação bilateral de C1-C2, foram introduzidos parafusos às massas laterais atlantoaxial bilateralmente. Utilizaram-se novamente fios K de 2,0 mm colocados cerca de 3mm a 4 mm lateralmente à linha média da odontoide, lateral à ponta do parafuso central na vista AP, e no mesmo ponto de entrada do que o parafuso à odontoide na vista lateral. A direção é inclinada de 20° a 25° lateralmente no plano frontal, e de 30° a 40° dorsalmente no plano sagital. Os fios K de 2,0 mm foram introduzidos na massa lateral de C1 logo abaixo da articulação occipitocervical. Então, dois parafusos canulados de 3,5 mm com rosca curta foram inseridos após a medição.

Após a cirurgia, radiografias da coluna cervical e TAC cervical foram realizadas para confirmar o posicionamento dos parafusos ( Figs. 3 e 4 ). Foi aplicado um colar cervical durante seis semanas e, depois, duas semanas de desabituação ao colar com períodos alternados de uso.

**Fig. 3:** Radiografia da coluna cervical nas incidências AP ( **A** ) e lateral ( **B** ).

**Fig. 4:** Tomografia axial computadorizada pós-operatória que mostra alinhamento correto dos parafusos nos planos axial ( **A** ), coronal ( **B** ) e sagital ( **C** ). Reconstrução 3D ( **D** ).

Um ano após cirurgia, o doente encontra-se extremamente satisfeito, sem dores ou défices neurológicos, tendo regressado aos níveis de atividade prévios à lesão. Como esperado, o doente apresenta uma diminuição da rotação cervical, com 35° de rotação cervical para cada lado. O estudo radiológico ao ano mostra consolidação da fratura ( Fig. 5 ).

**Fig. 5:** Tomografia axial computadorizada aos 12 meses de pós-operatório, que mostra consolidação de fratura e alinhamento correto dos parafusos nos planos axial ( **A** ), coronal ( **B** ) e sagital ( **C** ). É possível observar um ligeiro grau de osteólise em volta dos parafusos transarticulares, de acordo com o esperado por não haver artrodese, tal como referido na discussão.

## Discussão

A fratura de C2 é a lesão cervical mais comum na população idosa, sendo a fratura de tipo II de Anderson-D'Alonzo 8 o subtipo mais comum. Na população idosa, esta lesão constitui 60% a 80% das fraturas de C2, o que torna este um problema cada vez mais prevalente, dado o envelhecimento progressivo da população. 2

Quanto às fraturas de tipos I e III de Anderson-D'Alonzo, 8 existe um consenso com relação ao tratamento conservador. No entanto, o tratamento das fraturas de tipo II é controverso, especialmente na população idosa. 1

Nos casos desta lesão na população idosa, existe a opção de tratamento conservador, fusão por via posterior, ou fixação por via anterior. O tratamento conservador está associado a maiores taxas de pseudoartrose e inferior qualidade de vida. 5 As técnicas de fusão atlantoaxial posterior estão bem estabelecidas, e permitem excelentes resultados com altas taxas de fusão, mas levam a uma disseção cirúrgica mais agressiva e a um maior risco de lesão da artéria vertebral. A abordagem por via anterior tem os benefícios de o doente permanecer na posição supina, e de a disseção cirúrgica ser realizada mediante um espaço virtual, com menor agressão. 1 5 A fixação anterior é biomecanicamente comparável à fixação posterior, e uma alternativa válida a esta técnica. 10

Tendo em consideração a população idosa que frequentemente apresenta múltiplas comorbilidades médicas e risco anestésico alto, uma menor agressão cirúrgica tem benefícios claros.

Os autores admitem também que, mesmo não obtendo artrodese, a fixação transarticular bilateral entre as massas laterais C1-C2 pode garantir uma fixação estável o suficiente, não havendo relatos de falência destas fixações mesmo na ausência de artrodese consumada. Na população geriátrica, pensamos que esta técnica de fixação menos agressiva deve ser vista como primeira linha em casos de instabilidade traumática C1-C2. Entendemos que na população geriátrica, com a fixação com parafusos transarticulares anteriores, a artrodese não é absolutamente necessária, sendo esta estabilização habitualmente suficiente para garantir bons resultados clínicos, com reduzidos riscos de falência. No entanto, é expectável, precisamente pela ausência de artrodese, o aparecimento de algum grau de osteólise, sobretudo em volta dos parafusos transarticulares, tal como é visível na Fig. 5 .

Chamamos a atenção para o fato de que são necessários mais estudos para avaliar a eficácia desta fixação numa população mais jovem, dada a expectativa de vida e o nível funcional superiores, podendo estes pacientes ter um maior risco de falência destes parafusos transarticulares nas situações em que não se observa evolução para artrodese. Consideramos assim esta técnica ideal para pacientes geriátricos com instabilidade C1-C2 traumática; no entanto, não a utilizamos habitualmente por este motivo em pacientes jovens, preferindo técnicas que incluem mais gestos favorecedores de artrodese, nomeadamente fixações posteriores associadas a cruentação e aplicação local de enxerto ósseo. A osteossíntese da apófise odontoide é recomendada somente quando o traço de fratura é perpendicular ao parafuso de osteossíntese, sendo apenas nestes casos que optamos pela sua osteossíntese. Em casos em que o traço de fratura não é favorável a osteossíntese, após redução da fratura e luxação C1-C2, optamos apenas pela fixação transarticular anterior.
